# Medical insurance integration improves migrant workers’ employment quality: Evidence from China’s URRMI reform

**DOI:** 10.3389/fpubh.2025.1660023

**Published:** 2025-09-11

**Authors:** Yixin Gao, Wenguang Yu, Xinran Yu, Yujuan Huang

**Affiliations:** ^1^School of Statistics and Mathematics, Shandong University of Finance and Economics, Jinan, China; ^2^School of Insurance, Shandong University of Finance and Economics, Jinan, China; ^3^China Life Insurance Company Limited Shandong Branch, Jinan, China; ^4^School of Transportation and Logistics Engineering, Shandong Jiaotong University, Jinan, China

**Keywords:** Urban-Rural Resident Medical Insurance (URRMI), employment quality, migrant workers, mediation mechanism, social protection, health insurance

## Abstract

This study employs a staggered difference-in-differences approach combined with propensity score matching to evaluate the impact of the Urban-Rural Resident Medical Insurance (URRMI) integration on the employment quality of rural–urban migrant workers. Drawing on data from the 2014–2020 waves of the China Family Panel Studies (CFPS), the analysis constructs a multidimensional employment quality index encompassing wage income, social protection, job stability, and working hours. Mediation analysis identifies two key transmission channels: reduced out-of-pocket medical expenses and improved health status. The empirical results show that URRMI significantly enhances employment quality, with particularly notable improvements in wages, social protection coverage, and employment stability. Heterogeneity analysis further indicates that the policy effects are more pronounced among older workers, in eastern provinces, and within the primary and secondary sectors. These findings contribute new empirical evidence on the labor market implications of social insurance reform and provide timely policy insights to support the transformation of rural migrant workers into “new urban citizens” in the context of China’s inclusive urbanization agenda.

## 1 Introduction

China’s urbanization rate has now exceeded 65%, with nearly 300 million rural migrant workers serving as a vital force in urban economic development. These workers are registered under the rural hukou system but employed in urban non-agricultural sectors. Yet, improving their employment quality remains constrained by structural barriers, particularly the fragmented healthcare insurance system shaped by urban–rural dualism. For years, institutional fragmentation has led to a “covered but unprotected” dilemma, where health insurance fails to function as an effective safety net and inadvertently reinforces labor market segmentation.

China’s basic medical insurance system has historically evolved along fragmented lines, shaped by employment status, hukou category, and regional fiscal capacity. By the early 2000s, three parallel schemes coexisted: the Urban Employee Basic Medical Insurance (UEBMI), the Urban Resident Basic Medical Insurance (URBMI), and the New Rural Cooperative Medical Scheme (NRCMS). These systems differed in financing sources, benefit levels, and administrative oversight (1, 2). Migrant workers, though employed in cities, were generally enrolled in the NRCMS due to their rural hukou, facing limited benefits, poor portability, and dual-enrollment burdens (3–5). Empirical studies have shown that this fragmented structure reduces healthcare utilization and increases financial risk among mobile populations (2, 6, 54). This institutional arrangement further exacerbates the vulnerability of migrant workers in the labor market.

In 2016, the State Council initiated a national reform to integrate URBMI and NRCMS into the Urban-Rural Resident Medical Insurance (URRMI) system. This reform aimed to standardize financing, expand risk pooling, and promote equitable access to care (4, 7). It also sought to enhance cross-region benefit access, a particularly pressing issue for migrant workers navigating urban labor markets (6).

Within this framework, different regions adopted integration approaches tailored to local conditions, among which provincial pooling represents a higher-level model. This approach extends the geographic scope of risk pooling, strengthens the capacity for fund reallocation, and unifies institutional parameters such as the reimbursement list, reimbursement rates, deductibles, and ceilings, thereby improving protection levels and ensuring consistent entitlements across regions.

By raising reimbursement rates for groups previously covered by NRCMS, narrowing benefit gaps across regions, and increasing the fund’s ability to absorb high medical costs, such unification directly reduces the share of expenses paid out-of-pocket by enrollees. In addition, centralized procurement and harmonized payment procedures help lower price disparities and eliminate extra costs when seeking care outside one’s locality.

For migrant workers, coverage status is typically determined by the policies of their place of household registration. Once the registered province completes the integration and the individual was previously enrolled in either URBMI or NRCMS, they are automatically transferred into the unified URRMI system. The gradual implementation of provincial pooling enables migrant workers who move across cities within a province to receive care under the same reimbursement rates and claims processing rules, reducing institutional barriers and financial burdens. To some extent, this helps mitigate disadvantages in healthcare accessibility and benefit levels caused by the historical urban–rural divide, thereby contributing to the alleviation of structural inequality and excessive labor among migrant workers (8, 9).

Since the launch of URRMI, a growing body of research has examined its policy effects on healthcare utilization, subjective well-being, and financial protection (2, 10–13). However, less attention has been paid to how integration shapes employment outcomes, especially for migrant workers who continue to face structural disadvantages. This highlights the need to include employment quality in assessments of welfare integration, particularly salient given that employment quality has emerged as a central concern in social policy design for promoting inclusive growth and reducing inequality.

This shift has prompted scholars to move beyond employment status alone and examine the multidimensional conditions that constitute job quality. The International Labour Organization (ILO), through its Decent Work agenda launched in 1999, defines employment quality through four foundational components. These include job creation, rights at work, social protection, and social dialogue. Building on this framework, both policymakers and researchers have increasingly called for rigorous empirical standards to assess job quality (14–16). In response, recent studies have constructed multidimensional indicators that combine objective factors, such as income, job stability, working hours, and training opportunities, with subjective evaluations of autonomy, security, and work-life balance (17–22).

Moving from measurement to determinants, the factors shaping it operate at multiple levels. At the macro level, globalization and the digital economy have restructured labor demand, expanding high-skill formal employment while also accelerating the growth of low-security informal work. These shifts create new opportunities but simultaneously weaken labor protections and deepen employment segmentation. Institutional reforms, especially in welfare and healthcare, have further altered labor market incentives and reshaped employment choices, complicating employment relations (23–26). At the meso level, organizational practices and institutional segmentation significantly shape employment outcomes. Firm size, ownership type, and regulatory compliance affect access to formal contracts, social insurance, and labor protections. Migrant and informal workers are often concentrated in sectors characterized by subcontracting, labor dispatch, and weak unionization—structural features that jointly suppress job quality and reinforce inequality (27–30). At the micro level, education, gender, health, and social capital influence not only employment access but also job quality. Empirical studies consistently find that women, rural-origin workers, and those with lower education levels are more likely to face low wages, heavy workloads, and unstable working conditions (31, 32). In China, the hukou system continues to restrict rural migrants’ access to urban services such as health insurance and labor protections, thereby limiting their upward mobility in the labor market (3, 4, 33, 34). Consequently, employment quality is shaped not only by market dynamics but also by institutional boundaries and unequal access to welfare (35, 36).

In China, discussions around “high-quality and adequate employment” have appeared more frequently in recent policy discourse. Several studies have developed multidimensional indicators combining income, social security coverage, labor contract status, work intensity, and job satisfaction to evaluate employment quality in different populations (2, 37, 38). However, existing frameworks overlook important empirical dimensions, especially for rural-to-urban migrant workers who occupy a crucial yet often neglected position in China’s labor market. Like many informal workers in developing countries, they encounter structural constraints such as short-term contracts, limited social security, and frequent job mobility. Moreover, fragmented institutional arrangements and inconsistencies in regional data standards have long hindered accurate assessment of migrant workers’ employment quality (39, 40). The 2016 integration of the Urban–Rural Resident Medical Insurance (URRMI) system, hereafter referred to as the URRMI reform, unified urban and rural schemes, improved data comparability and policy traceability across regions, and provided a valuable context for analyzing how social protection influences employment outcomes.

## 2 Stylized facts

This section builds on the preceding literature review to outline recent developments in China’s URRMI reform and the employment conditions of migrant workers. By documenting statistical trends and institutional changes, it aims to provide a factual and policy context for the empirical analysis that follows.

### 2.1 The evolution of integration of urban and rural basic medical insurance systems

**Figure 1:**
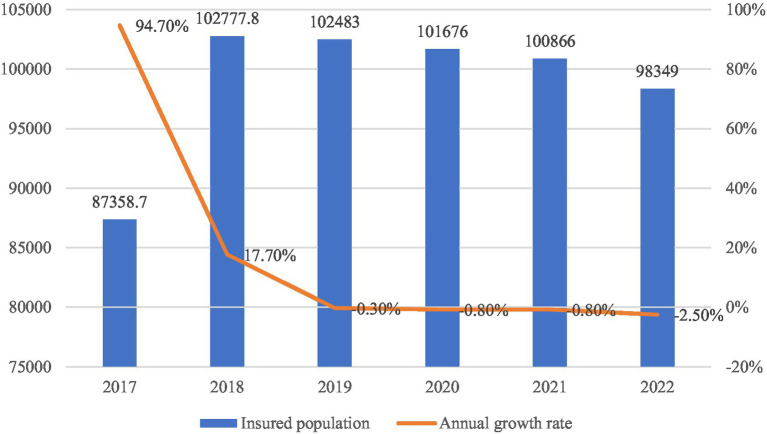
URBMI enrollment (2017–2022, in 10,000 s). Data source: China Statistical Yearbook (2017–2018) & national healthcare security development statistical bulletin (2019–2022).

Since 2016, China has gradually merged the URBMI and the NRCMS into a unified URRMI system. As shown in Figure 1, the number of URRMI enrollees surged to 873.59 million in 2017, with a year-on-year growth rate of 94.7%. This initial surge was largely driven by changes in statistical classification and the concentrated expansion of coverage during the early stage of integration. In subsequent years, enrollment remained high but growth slowed, even turning negative after 2019. By 2022, total enrollment declined to 983.49 million, marking a decrease of 15.17 million from the previous year. This decline reflects the elimination of duplicate enrollments and broader demographic transitions, including population aging and changing migration dynamics. While extensive coverage has been achieved, the system is now shifting toward deeper institutional integration, laying the groundwork for more equitable inclusion of mobile populations such as migrant workers.

**Figure 2:**
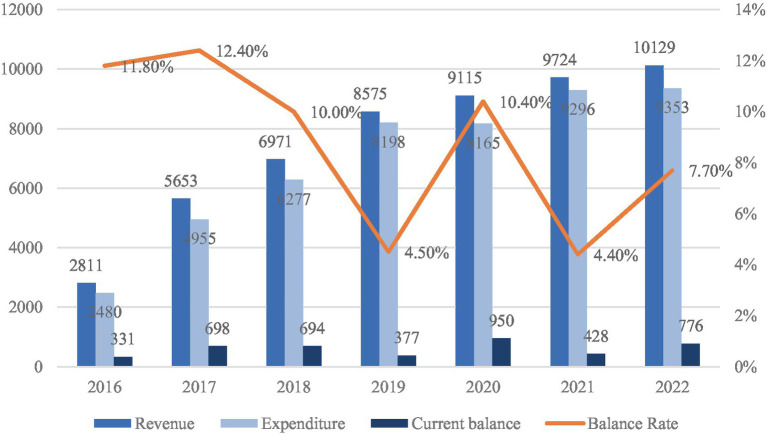
URBMI fund revenue and expenditure (2016–2022, in 100 million). Data source: national healthcare security development statistical bulletin (2016–2022).

The fiscal dynamics of the URRMI system are illustrated in Figure 2. Between 2016 and 2022, healthcare fund revenues expanded from RMB 281.1 billion to RMB 1,012.9 billion, while expenditures increased from RMB 248.0 billion to RMB 935.3 billion. Although both revenue and spending grew steadily, the fund’s surplus ratio exhibited a downward trend. This trend reflects growing pressure from rising medical demand, aging, and uneven regional development. In response, deeper pooling and more coordinated funding are needed to maintain system stability. For migrant workers, who often move across regions, a stable and unified system is essential to ensure consistent access to care and reduce employment-related vulnerability.

**Figure 3:**
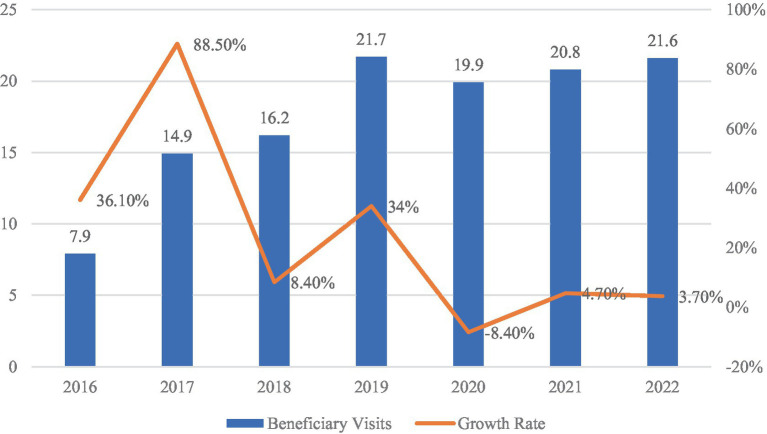
URBMI beneficiary visits (2016–2022, in person-times). Data source: national healthcare security development statistical bulletin (2016–2022).

**Figure 4:**
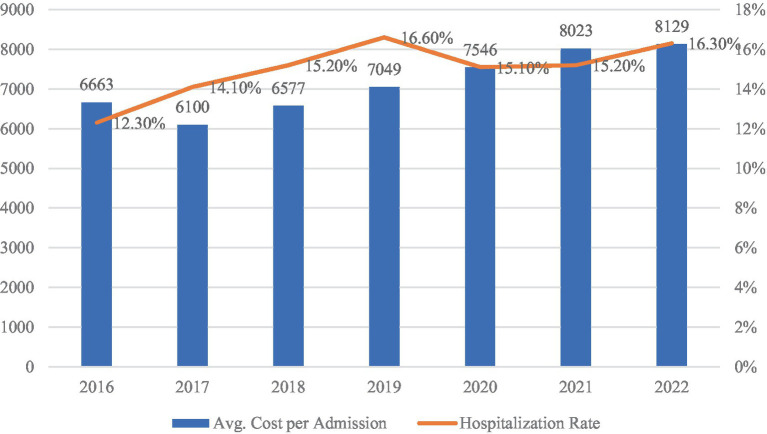
URBMI average inpatient cost per admission (2016–2022, in CNY). Data source: national healthcare security development statistical bulletin (2016–2022).

Figures 3, 4 illustrate trends in the utilization of urban–rural resident medical insurance, measured, respectively, by the number of treatment episodes and the average inpatient cost per admission. As shown in Figure 3, total treatment episodes increased from 790 million in 2016 to 2.16 billion in 2022, with a peak growth rate of 88.5% in 2017. Although some fluctuations occurred thereafter, the overall trajectory remained upward. Figure 4 shows that average inpatient costs rose steadily from RMB 6,663 in 2016 to RMB 8,129 in 2022. These developments indicate a continuous expansion in both coverage and frequency of healthcare service use as institutional integration advanced. As one of the key populations covered by the system, migrant workers have also seen increased access to medical services during periods of urban employment. However, whether this expansion in service utilization translates into improved employment quality cannot be determined from aggregate indicators alone. Its specific impact requires empirical validation based on micro-level data.

### 2.2 Employment conditions and challenges among migrant workers

**Figure 5:**
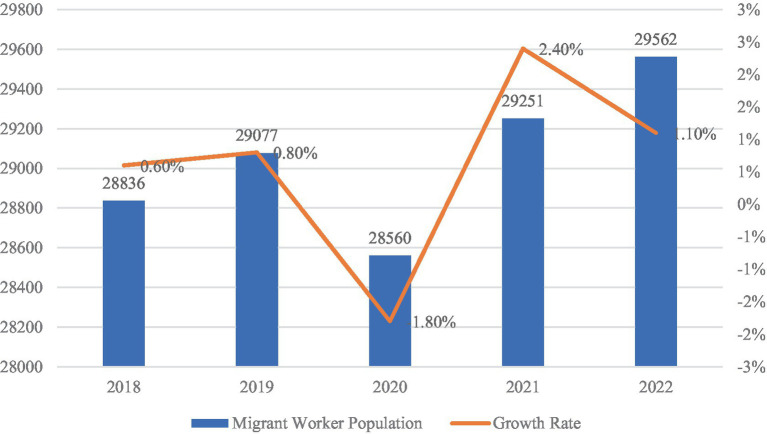
Migrant worker population and growth rate (2018–2022, in 10,000 persons). Data source: migrant workers monitoring survey report (2018–2022).

Migrant workers play a central role in balancing labor allocation between urban and rural areas. Shifts in their size and structure directly affect labor market stability and the responsiveness of public policies. As shown in Figure 5, the total number of migrant workers increased from 288.36 million in 2018 to 295.62 million in 2022. A temporary decline occurred in 2020, when the number dropped to 285.60 million, largely due to pandemic-related disruptions. However, the population rebounded in 2021 and 2022, returning to a trajectory of gradual growth. This sustained scale highlights the continued structural importance of migrant labor to China’s economic development.

Sectoral data from Table 1 indicate that migrant workers are primarily concentrated in the secondary and tertiary industries. In 2022, 47.8% were employed in the secondary industry, with 27.4% in manufacturing and 17.7% in construction. The tertiary industry absorbed 51.7% of migrant workers, with employment distributed across wholesale and retail trade (12.5%), residential services, repair, and other services (11.9%), accommodation and catering services (6.1%), and transportation, storage, and postal services (6.8%). While employment growth was generally stable across sectors, construction experienced a year-on-year decline of 1.3%. This pattern underscores the continued concentration of migrant labor in labor-intensive, low value-added segments of the economy.

**Table 1:** Employment distribution of migrant workers by industry (2022, Unit: %).

Industry	Distribution in 2022	Growth rate
Primary Industry	0.5	0.0
Secondary Industry	47.8	−0.8
Manufacturing	27.4	0.3
Construction	17.7	−1.3
Tertiary Industry	51.7	0.8
Wholesale and Retail Trade	12.5	0.4
Transportation, Storage, and Postal Services	6.8	−0.1
Accommodation and Catering Services	6.1	−0.3
Residential Services, Repair, and Other Services	11.9	0.1

Data source: migrant workers monitoring survey report 2022.

**Figure 6:**
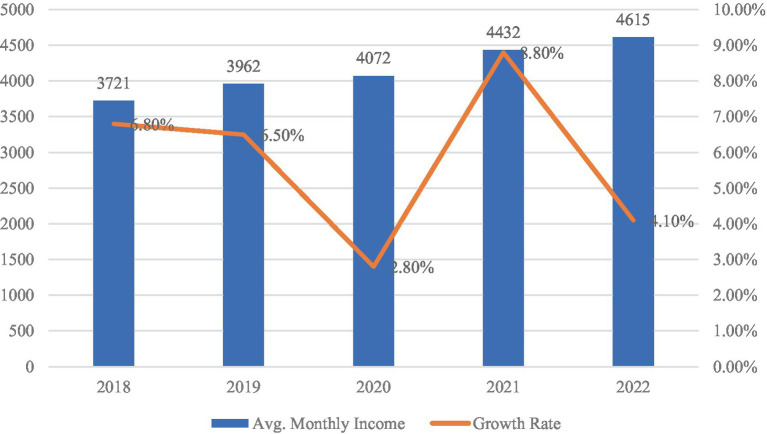
Monthly average income of migrant workers (2018–2022, in CNY). Data source: migrant workers monitoring survey report (2018–2022).

According to Figure 6, the average monthly wage of migrant workers rose from RMB 3,721 in 2018 to RMB 4,615 in 2022. While nominal wages increased steadily overall, the growth rate fluctuated across years, reaching a low in 2020 and rebounding sharply in 2021. This volatility suggests that income levels among migrant workers are sensitive to broader macroeconomic trends.

In sum, migrant employment has remained relatively stable in terms of overall size and sectoral composition, with gradual improvements in income. However, challenges in employment quality persist, particularly due to limited access to social protection. The following analysis explores whether the URRMI reform has significantly influenced the labor market position of migrant workers, and whether improved access to healthcare services contributes to more stable and regulated employment conditions.

## 3 Data and method

### 3.1 Mechanism and hypotheses

Therefore, this study links these literatures by examining how URRMI reform influences the employment quality of migrant workers. Based on key features of migrant labor conditions in China, we construct an employment quality index that covers income, social protection, job stability, and work intensity. Leveraging the staggered rollout of URBMI–NRCMS integration, we employ a staggered difference-in-differences (Staggered DID) approach to identify causal effects and investigate heterogeneity and mediating channels.

Theoretical insights suggest two primary pathways through which insurance integration may influence employment quality. First, liquidity constraints significantly shape labor behavior. Migrant workers facing high medical cost uncertainty often accept low-wage, unstable jobs to maintain cash flow (31, 41, 42). By reducing out-of-pocket healthcare expenses, URRMI reform alleviates precautionary labor supply, enabling transitions to less intensive and more stable employment. Second, health is a fundamental determinant of labor capacity. Better health correlates with higher productivity and longer job tenure (43, 44). Expanded insurance coverage and service access under URRMI enhance migrant workers’ physical well-being, thereby strengthening labor market attachment and quality (45–50).

Moreover, substantial differences may exist within the migrant worker population in terms of the benefits gained from medical insurance integration. First, age differences affect not only health status but also labor capacity and employment stability. Older migrant workers, having often engaged in high-intensity physical labor for extended periods, face higher health risks and greater medical needs; as a result, they may experience more pronounced reductions in healthcare burdens and improvements in employment quality after the reform. Second, significant disparities exist in regional development levels and public resource provision. While eastern coastal regions generally have better healthcare accessibility and more diverse labor markets, migrant workers in central and western regions may benefit more from the enhanced level of protection brought about by integration. Finally, there is notable variation in the types of industries in which migrant workers are employed. Some industries are characterized by high labor intensity and elevated risks of occupational injury, whereas others offer relatively stable positions and higher skill requirements. These differences imply that the impact of medical insurance integration on employment quality may vary across sectors. Given these characteristics, it is necessary to examine effect heterogeneity among different subgroups in the empirical analysis.

Building on these mechanisms, we propose three hypotheses.

*H1:* Urban–rural medical insurance integration significantly improves the employment quality of rural–urban migrant workers.

*H2:* The effect of insurance integration on employment quality varies across subgroups defined by age, region, or employment sector.

*H3:* The impact of insurance integration on employment quality is mediated through reductions in healthcare burden and improvements in individual health.

### 3.2 Data sources and variable description

The primary data sources for this study include: (1) Individual characteristics and employment quality data of migrant workers from four waves (2014, 2016, 2018, and 2020) of the China Family Panel Studies (CFPS); (2) Regional macroeconomic variables from the China Statistical Yearbook and provincial Statistical Communiqués on National Economic and Social Development; (3) Policy implementation data for the URRMI reform obtained from local government documents. Following the definition in the 2022 Migrant Workers Monitoring Survey Report, the study population consists of individuals with agricultural household registration who engaged in local non-agricultural employment or worked outside their hometown for at least 6 months annually, aged 16–60 (male) or 16–55 (female).

The empirical design incorporates three categories of variables. The key explanatory variable is URRMI reform ( policy ), coded as 1 if a migrant worker resided in a province that had implemented the integration policy by year t , and 0 otherwise. The outcome variable is a composite index of employment quality, consisting of four standardized dimensions: wage income ( wage ), social protection ( benefits ), job stability ( stability ), and work intensity (intensity). Wage income is calculated as the natural logarithm of individual monthly earnings. Social protection is based on the number of enrolled social insurance items (pension, medical, work injury, maternity, and unemployment), scored as the sum of items reported. Job stability is captured by a binary variable indicating whether the worker signed a formal labor contract (1 = yes, 0 = no). Work intensity is measured by total weekly working hours. All four components were standardized following Equation 1, with work intensity being reverse-standardized (i.e., one minus the normalized value).

The standardization formula is as follows:

(1) $$x_{\mathrm{ij}}^{\mathrm{nor}}=\frac{\left(x_{\mathrm{ij}}-\min_{j}\right)}{\left(\max_{j}-\min_{j}\right)}$$

where $x_{\mathrm{ij}}^{\mathrm{nor}}$ is the normalized value for individual i on dimension j ; $\max_{j}$ and $\min_{j}$ represent the maximum and minimum values of dimension j , respectively. The final employment quality index is calculated as the unweighted average of the four standardized components, scaled to a 0–10 range, with higher values indicating better employment quality.

Control variables are selected with reference to prior research on rural–urban labor migration, informal employment, and social integration. At the individual level, we control for age ( age ), gender ( gen ), education level ( edu ), marital status ( mar ), household size ( fam ), migration distance ( com ), and promotion status ( pro ). At the regional level, we include openness to trade ( ope ), public service provision ( pub ), and urbanization rate ( urb ). Details on variable definitions are presented in Table 2.

**Table 2:** Variables and definition.

Variable type	Variable name	Variable definition
Dep Var	Employment Quality ( quality )	Composite index (0–10) combining wage income, social protection, job stability, and work intensity dimensions
Indep Var	Policy Integration ( policy )	Binary indicator for urban–rural medical insurance integration (1 = implemented, 0 = otherwise)
	Age ( age )	ln(age in years)
	Gender ( gender )	1 = male, 0 = female
	Education level ( edu )	1 = high school+, 0 = less than high school
	Marital Status ( mar )	1 = married/cohabiting/divorced/widowed, 0 = single
	Family Size ( family )	ln(number of household members)
	Commute Time ( com )	Daily round-trip commute minutes
	Promotion Satisfaction ( pro )	5-point Likert scale satisfaction with advancement opportunities
	Trade Openness ( open )	Regional (exports+imports)/GDP ratio
	Public Service ( public )	Government expenditure/GDP ratio
	Urbanization Rate ( urban )	% population living in urban areas

Descriptive statistics are presented in Table 3. Among the sampled migrant workers, 49.3% were covered by the URRMI reform. The employment quality index displays substantial variation, with social protection coverage reaching only 26.1%, highlighting the overall weakness of welfare access among migrant workers. The work intensity indicator shows an approximately symmetric distribution, suggesting a balanced spread of labor intensity across the sample. The mechanism variables, health status ( health ) and medical expenditure ( cost ), will be further examined in subsequent mediation analysis. Overall, the staggered rollout of URRMI across regions and time justifies the use of a staggered DID strategy, while variation in employment quality ensures sufficient statistical power for policy effect identification. Based on this, we evaluate the impact of insurance integration, controlling for relevant covariates.

**Table 3:** Descriptive statistics.

Var	Mean	SD	Min	Max	Med
quality	4.334	1.991	0.013	8.354	3.315
policy	0.493	0.500	0	1	0
wage	0.689	0.0860	0	1	0.698
benefits	0.261	0.399	0	1	0
stability	0.456	0.498	0	1	0
intensity	0.328	0.103	0.005	1	0.333
age	3.444	0.303	2.773	4.094	3.401
gen	0.551	0.497	0	1	1
edu	0.256	0.437	0	1	0
mar	0.718	0.450	0	1	1
fam	1.172	0.655	0	2.833	1.386
com	2.613	1.017	0	5.485	2.773
pro	3.154	0.901	1	5	3
ope	0.294	0.297	0.008	1.216	0.142
pub	1.727	0.749	0.837	11.940	1.537
urb	0.584	0.113	0.338	0.893	0.564
health	2.758	1.051	1	5	3
cost	5.869	1.922	0	12.210	5.994

### 3.3 Model specification

The baseline specification is specified as follows:

(2) $$\text{qualit}y_{ict}=\alpha +\text{βpolic}y_{\mathrm{ict}}+\phi X_{\mathrm{ict}}+\lambda_{t}+\gamma_{c}+\varepsilon_{\mathrm{ict}}$$

where the dependent variable $\text{qualit}y_{\mathrm{ict}}$ measures the employment quality of migrant worker i in region c during year t . The key explanatory variable $\text{polic}y_{\mathrm{ict}}$ indicates whether the individual was covered by the URRMI reform in a given year. The model includes a vector of control variables $X_{\mathrm{ict}}$ , year fixed effects $\lambda_{t}$ and region fixed effects $\gamma_{c}$ . The error term $\varepsilon_{\mathrm{ict}}$ captures all other unobserved factors affecting employment quality. The coefficient β captures the average treatment effect of the URRMI reform. This specification exploits the staggered rollout of the URRMI reform across regions and over time to estimate its causal impact on employment quality. The coefficient ϕ captures the effects of controls, and α is the constant.

## 4 Results

### 4.1 Baseline results

Following Equation 2, we estimate the effect of urban–rural medical insurance integration on the employment quality of migrant workers using a staggered DID approach. The baseline regression results are presented in Table 4. Columns (1) to (3) sequentially incorporate individual-level covariates and regional-level controls. Robust standard errors are clustered at the regional level.

**Table 4:** Baseline results.

var	(1)	(2)	(3)
policy	0.175^**^	0.192^**^	0.310^***^
	(0.089)	(0.084)	(0.092)
age		−0.587^***^	−0.585^***^
		(0.090)	(0.090)
gen		0.311^***^	0.307^***^
		(0.042)	(0.042)
edu		1.470^***^	1.464^***^
		(0.058)	(0.058)
mar		0.297^***^	0.294^***^
		(0.062)	(0.062)
fam		−0.187^***^	−0.176^***^
		(0.035)	(0.035)
com		0.093^***^	0.093^***^
		(0.021)	(0.021)
pro		0.045^*^	0.045^*^
		(0.023)	(0.023)
ope			−0.542
			(0.492)
pub			−0.349^**^
			(0.143)
urb			−1.531
			(2.826)
cons	4.247^***^	5.334^***^	6.919^***^
	(0.049)	(0.304)	(1.519)
Year	Yes	Yes	Yes
Region	Yes	Yes	Yes
N	6,985	6,985	6,985
$R^{2}$	0.162	0.259	0.260

***Indicates statistical significance at the 10%, 5%, and 1% levels, respectively.

The results indicate a consistently positive and statistically significant effect of the URRMI reform across specifications. The estimated coefficient increases from 0.175 in Column (1) to 0.310 in Column (3), suggesting that the policy effect is robust to the inclusion of additional covariates.

Regarding the control variables, the direction and significance of most estimates align with theoretical expectations. Age is negatively associated with employment quality, which may reflect the physical demands of migrant labor that disproportionately affect older workers. Male workers exhibit significantly higher employment quality than females, indicating persistent gender disparities in labor market outcomes. Education has a strong positive effect, consistent with the role of human capital in enhancing employment outcomes. Marital status also shows a positive association, potentially capturing employment stability among married individuals. In contrast, larger household size is negatively correlated with employment quality, possibly due to increased caregiving responsibilities or reduced labor mobility.

At the regional level, public service expenditure is negatively correlated with employment quality. This finding may stem from hukou-based exclusion, whereby increased investment in public goods disproportionately benefits local residents, further marginalizing migrant workers.

### 4.2 Robustness verification: PSM-DID approach

To further verify the robustness of the baseline results, this section employs a Propensity Score Matching Difference-in-Differences (PSM-DID) approach to mitigate potential estimation bias arising from sample selection. We first report the results under the nearest-neighbor matching specification, and then conduct sensitivity checks using alternative matching algorithms to strengthen the credibility of the estimated effects.

#### 4.2.1 Baseline estimation

To address potential selection bias in policy implementation, this study employs PSM-DID approach as a robustness check. By constructing a counterfactual framework through matching, this method helps mitigate systematic differences in observable characteristics between treated and control groups. As shown in Table 5, after 1:3 nearest-neighbor matching, the estimated coefficient of the insurance integration policy is 0.334 and remains statistically significant at the 1% level. The estimate is directionally consistent with the baseline results, supporting the robustness of the policy effect and indicating that the findings are unlikely to be driven by selection bias.

**Table 5:** PSM-DID baseline results.

var	Nearest neighbor matching	
	(1)	(2)
policy	0.285^***^	0.334^***^
	(0.099)	(0.101)
covariates	Yes	Yes
cons	4.179^***^	6.880^***^
	(0.050)	(1.718)
Year	Yes	Yes
Region	Yes	Yes
N	6,318	6,318
$R^{2}$	0.171	0.270

***Indicates statistical significance at the 1% level.

#### 4.2.2 Alternative matching algorithms

To assess whether the results are sensitive to the choice of matching algorithm, we re-estimate the treatment effects using radius caliper matching and kernel matching in addition to the baseline nearest-neighbor method. Table 6 summarizes the results across all specifications. The estimated effect of URRMI reform on the employment quality of migrant workers remains positive and statistically significant at the 1 percent level across all specifications, with coefficients consistently close to 0.334. The consistency of results across different matching strategies supports the robustness of the findings and indicates that the quality of matching is adequate, without evident issues of poor covariate balance or insufficient common support. These results enhance the credibility of the causal interpretation of the estimated treatment effect.

**Table 6:** Sensitivity analysis.

Var	Radius matching		Kernel matching	
	(1)	(2)	(3)	(4)
policy	0.285^***^	0.334^***^	0.285^***^	0.334^***^
	(0.099)	(0.101)	(0.099)	(0.101)
covariates	No	Yes	No	Yes
cons	4.179^***^	6.880^***^	4.179^***^	6.880^***^
	(0.050)	(1.718)	(0.050)	(1.718)
Year	Yes	Yes	Yes	Yes
Region	Yes	Yes	Yes	Yes
N	6,318	6,318	6,318	6,318
$R^{2}$	0.171	0.270	0.171	0.270

***Indicates statistical significance at the 1% level.

### 4.3 Placebo test

**Figure 7:**
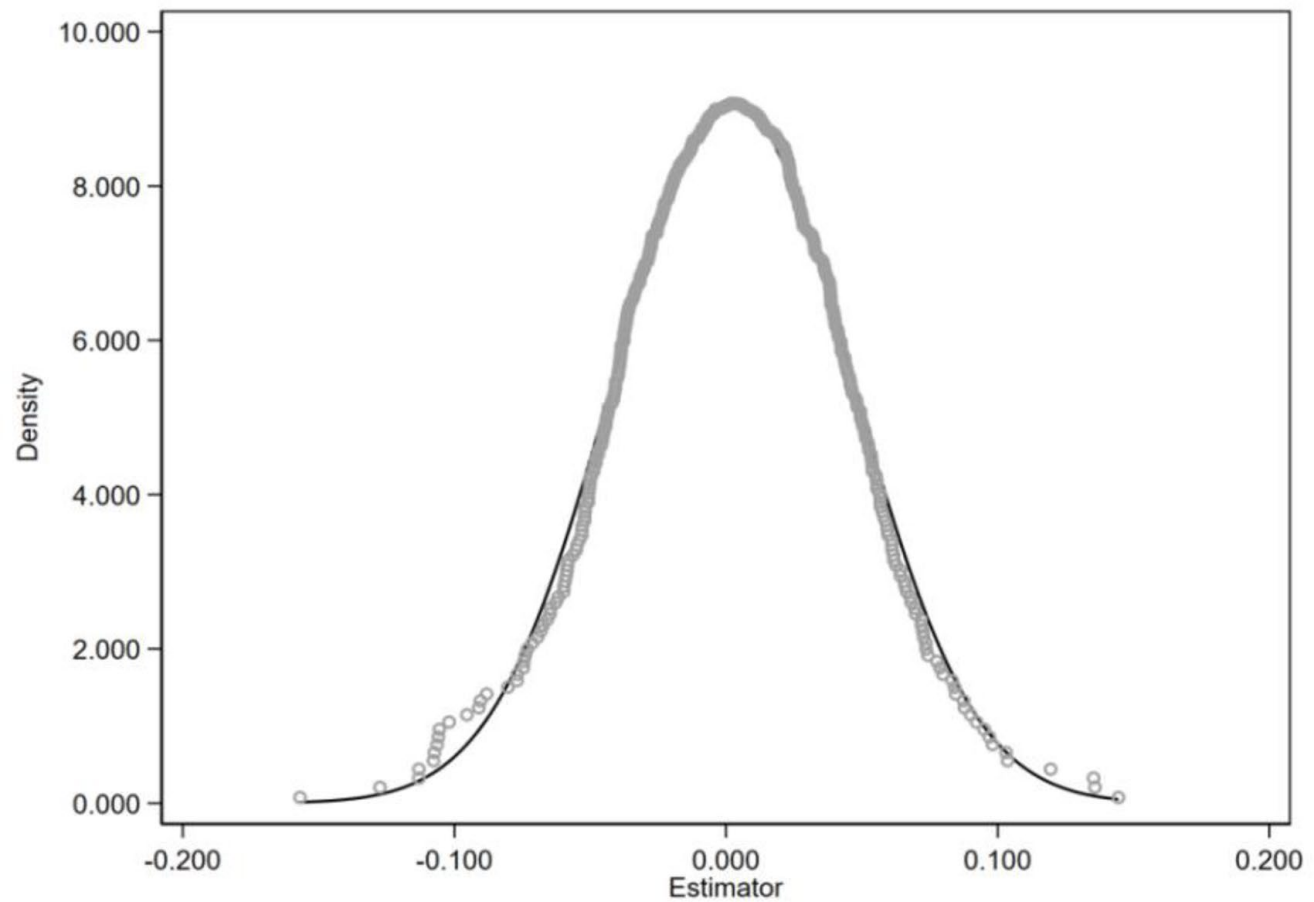
Placebo test.

To assess the reliability of the benchmark results, a placebo test was conducted by randomly assigning treatment status 500 times and re-estimating the model. As shown in Figure 7, the vast majority of placebo estimates are concentrated around zero. The distribution of the simulated coefficients exhibits no significant deviation, while the actual estimated effect lies clearly outside this range. This indicates that the observed effect is unlikely to be driven by model specification or sampling error, thus supporting the identification strategy and confirming the robustness of the findings.

### 4.4 Robustness extensions

#### 4.4.1 Alternative data and outcome specifications

To further assess the robustness of the findings, this section conducts additional tests using alternative data sources and outcome definitions. First, the policy implementation time is adjusted to January 2017 to better reflect the actual onset of large-scale pilot programs. Second, a subjective measure of job satisfaction is added to the existing four objective dimensions, to construct a broader employment quality index. Using the 2016 and 2018 waves of the China Labor-force Dynamics Survey (CLDS), the results remain consistent in direction and statistically significant across specifications, as reported in Table 7 [columns (1) and (2)].

**Table 7:** Alternative data and outcome specifications.

var	(1)	(2)	(3)	(4)
policy	0.463^***^	0.387^***^	0.292**	0.456**
	(0.097)	(0.093)	(0.123)	(0.184)
covariates	No	Yes	Yes	Yes
cons	3.753^***^	2.355^***^	3.813***	4.625***
	(0.023)	(0.152)	(0.027)	(0.271)
Year	Yes	Yes	Yes	Yes
Region	Yes	Yes	Yes	Yes
N	5,421	5,421	6,985	5,421
$R^{2}$	0.004	0.089	0.059	0.073

**,***Indicates statistical significance at the 5% and 1% levels, respectively.

Building on the above expansion of the outcome measure, and to ensure both comprehensive indicator coverage and analytical robustness, we further apply the entropy-weight method to the four objective dimensions in the CFPS data and to all five dimensions (including job satisfaction) in the CLDS data. The entropy-weight method assigns weights according to each dimension’s information entropy, thereby mitigating the influence of any single noisy indicator. The regression results for these entropy-weighted indices appear in Table 7, columns (3) and (4), with both sets of estimates aligning in sign with the main analysis and remaining statistically significant, further reinforcing the positive impact of the URRMI reform on migrant workers’ employment quality.

#### 4.4.2 Heterogeneity Bias and robust estimation

Recent studies point out that traditional staggered DID models may suffer from bias when treatment effects vary across cohorts and over time (51, 52). Because individuals receive treatment at different points in time, the sample can be split into multiple cohorts. Earlier-treated groups (often called “bad controls”) may serve as controls for later-treated groups, yet their pre-treatment trends may have already shifted compared to later-treated or never-treated groups (“good controls”). This can lead to potential bias in the estimated effects. To address this issue, we employ diagnostic tools to assess the extent of heterogeneity bias and then adopt estimation strategies that are robust to such bias.

##### 4.4.2.1 Bacon decomposition

To check whether our results are significantly affected by time heterogeneity, we first use the Bacon decomposition to break the overall estimate into weighted averages across different comparison types. Table 8 shows that most of the weight comes from comparisons between treated and never-treated groups and between treated and not-yet-treated groups. Comparisons between early- and late-treated groups account for less than 5%. The average estimates across all four types are very close, suggesting that time heterogeneity is unlikely to have a meaningful impact on the overall results. To address this issue, we employ diagnostic tools to assess the extent of heterogeneity bias and then adopt estimation strategies that are robust to such bias.

**Table 8:** Bacon decomposition.

Type	Weight	Avg.estimate
Treat vs. Never	0.68	0.300
Treat vs. Not-yet	0.25	0.295
Early vs. Late	0.04	0.302
Late vs. Early	0.03	0.298

##### 4.4.2.2 Heterogeneity-robust estimation

Beyond the Bacon decomposition, researchers have developed several approaches to address heterogeneity bias in multi-period settings. Broadly, these can be grouped into three types. The first estimates cohort–time average treatment effects (CATT) and then aggregates them across both cohorts and time. This avoids using earlier-treated groups as controls, reducing bias risk, but may lose some observations and efficiency, and requires the presence of a “never-treated” group. The second, imputation-based methods, construct counterfactual outcomes for treated units using never-treated or not-yet-treated observations, offering higher efficiency but depending more on correct model specification. The third, stacking estimators, match treated units to never- or not-yet-treated units, create separate datasets, and then stack them for regression with fixed effects. This approach avoids “bad controls” but may involve repeated use of the same data. In our sample, a sizeable never-treated group is available, making the first approach suitable. We therefore apply the method proposed by Callaway & Sant’Anna (53). We can see from Table 9 that the estimates from all three specifications are close to the baseline regression. The event-study results show that the average effect before policy implementation (Pre_avg) is close to zero and statistically insignificant, further confirming the robustness of the policy effect.

**Table 9:** Heterogeneity-robust estimation.

Var	ATT (simple)	ATT (event)	ATT (calender)
Simple	0.296^**^		
	(0.092)		
Pre_avg		0.011	
		(0.045)	
Post_avg		0.327^***^	
		(0.093)	
CAverage			0.294^**^
			(0.087)

**, ***Indicates statistical significance at the 5% and 1% levels, respectively.

#### 4.4.3 Dimension-specific effects

The results in Table 10 reveal heterogeneous effects of the URRMI reform across employment quality dimensions. The strongest improvement appears in social protection, which directly reflects the institutional changes brought by the integration. Wage income and job stability also show statistically significant gains, which may reflect reduced precautionary savings or eased liquidity constraints after policy implementation. In contrast, the reform did not produce a significant effect on work intensity. On the one hand, although the policy reduced certain medical expenses, the extent of financial relief was relatively limited and insufficient to prompt migrant workers to substantially reduce their working hours. On the other hand, a large proportion of migrant workers are concentrated in labor-intensive sectors, where rigid working schedules and widespread piece-rate payment systems constrain individual control over working time. Moreover, due to income instability and limited social protection, workers often maintain high labor intensity as a precaution against future uncertainties, reflecting a structural tendency toward “overwork.” Additionally, this study uses weekly working hours as a proxy for work intensity. While useful, this measure may not fully capture non-time-related pressures such as psychological stress or work rhythm, potentially limiting the identification of policy effects.

**Table 10:** Effects of URRMI reform on different dimensions of employment quality.

var	(1)	(2)	(3)	(4)
	Wage income	Social protection	Job stability	Work intensity
policy	0.120^**^	0.280^***^	0.050^**^	−0.008
	(0.050)	(0.092)	(0.024)	(0.005)
age	−0.187^***^	−0.476^***^	−0.116^***^	0.006
	(0.049)	(0.090)	(0.024)	(0.005)
gen	0.442^***^	0.242^***^	0.020^*^	−0.016^***^
	(0.023)	(0.042)	(0.011)	(0.002)
edu	0.098^***^	1.674^***^	0.297^***^	0.054^***^
	(0.031)	(0.057)	(0.015)	(0.003)
mar	0.167^***^	0.203^***^	0.044^***^	−0.018^***^
	(0.034)	(0.062)	(0.016)	(0.004)
fam	−0.088^***^	−0.103^***^	−0.031^***^	0.012^***^
	(0.019)	(0.035)	(0.009)	(0.002)
com	0.017	0.157^***^	0.014^**^	0.010^***^
	(0.011)	(0.021)	(0.005)	(0.001)
pro	−0.006	0.064^***^	0.014^**^	0.009^***^
	(0.013)	(0.023)	(0.006)	(0.001)
ope	−0.297	−0.333	−0.094	0.031
	(0.268)	(0.491)	(0.130)	(0.028)
pub	−0.099	−0.505^***^	−0.033	−0.003
	(0.078)	(0.142)	(0.038)	(0.008)
urb	−0.088	−4.126	0.244	0.024
	(1.538)	(2.821)	(0.749)	(0.162)
cons	8.480^***^	4.983^***^	0.609	0.577^***^
	(0.827)	(1.516)	(0.403)	(0.087)
Year	Yes	Yes	Yes	Yes
Region	Yes	Yes	Yes	Yes
N	6,985	6,985	6,985	6,985
$R^{2}$	0.101	0.267	0.169	0.094

**, ***Indicates statistical significance at the 5% and 1% levels, respectively.

The estimates also shed light on how control variables relate to employment outcomes. The negative association between age and employment outcomes in wage, benefit, and stability dimensions suggests that older migrant workers face greater challenges in employment quality. The strong contribution of education points that workers with higher education levels are more likely to access policy benefits and actively participate in formal insurance systems. Gender differences in wage income suggest that occupational segregation may weaken policy effects for female workers. Commute distance shows stable and positive associations across multiple dimensions, whereas public service provision exhibits a significant negative correlation only with social protection. These findings suggest possible structural differences in policy effects across regions, underscoring the importance of examining regional heterogeneity.

### 4.5 Heterogenous analysis

#### 4.5.1 Age-based heterogeneity

In the China's No.1 Central Document for 2010, the State Council formally introduced the concept of the “new generation of rural migrant workers,” referring to individuals born in or after 1980 who hold rural household registration but engage in long-term non-agricultural employment. Considering the rising occupational vulnerability among workers over the age of 40 and intergenerational differences in labor market experiences, the sample is divided into a younger cohort (aged ≤40) and an older cohort (aged >40) for subgroup analysis.

Table 11 reports estimates from three specifications. Columns (1) and (2) present results for the younger and older cohorts, respectively. Column (3) includes both groups and adds an interaction term between the URRMI reform and age.

**Table 11:** Age-based Heterogeneity.

var	(1)	(2)	(3)
	Younger cohort	Older cohort	With interaction
policy∗age			0.057^**^
			(0.023)
policy	0.299^***^	0.424^**^	0.266^**^
	(0.110)	(0.165)	(0.115)
age	0.320^**^	−2.026^***^	−0.782^**^
	(0.157)	(0.372)	(0.360)
covariates	Yes	Yes	Yes
Year	Yes	Yes	Yes
Region	Yes	Yes	Yes
N	5,283	1702	6,985
$R^{2}$	0.254	0.205	0.101

**, ***Indicates statistical significance at the 5% and 1% levels, respectively.

The results show that the effect of URRMI reform on employment quality is significantly stronger for older migrant workers. This pattern is consistent with the institutional structure of China’s medical insurance system, which typically provides higher reimbursement rates and broader medication coverage for older enrollees. These findings suggest that the mechanisms linking insurance integration to employment outcomes may vary by age group. For older workers, the effect likely operates through direct reductions in out-of-pocket medical expenses. Among younger, relatively healthier individuals, the policy may influence employment quality more through enhanced job stability and reduced income insecurity.

#### 4.5.2 Region-based heterogeneity

To assess regional variation in the policy effect, we estimate separate regressions for the eastern, central, and western regions, while continuing to control for province fixed effects. In the subgroup regressions, the eastern region shows a clear and statistically significant policy effect, indicating stronger responsiveness to policy implementation in this region. We further estimate a pooled model with a policy–region interaction term. As we can see in Table 12, the interaction between the policy variable and an eastern region indicator is positive and statistically significant, confirming that the policy impact in the east is significantly larger than in other regions. This finding likely reflects the advantages of the east in institutional capacity, healthcare infrastructure, and enforcement mechanisms, which enhance the transmission of policy benefits to migrant workers. In contrast, weaker implementation capacity and limited medical accessibility in central and western regions may have constrained the effectiveness of the policy.

**Table 12:** Region-based heterogeneity.

var	(1)	(2)	(3)	(4)
	Eastern	Central	Western	With interaction
policy∗east				0.210^**^
				(0.106)
policy	0.542^***^	0.093	0.313	0.264^**^
	(0.142)	(0.277)	(0.211)	(0.127)
covariates	Yes	Yes	Yes	Yes
Year	Yes	Yes	Yes	Yes
Region	Yes	Yes	Yes	Yes
N	3,197	1755	2033	6,985
$R^{2}$	0.276	0.185	0.240	0.109

***Indicates statistical significance at the 10%, 5%, and 1% levels, respectively.

#### 4.5.3 Industry-based heterogeneity

Table 13 shows that the effect of the URRMI reform on employment quality differs significantly across sectors. The interaction regression suggests that the policy effect is stronger in non-tertiary industries, consistent with the results of the subgroup analysis.

**Table 13:** Industry-based heterogeneity.

Var	(1)	(2)	(3)
	Non-tertiary sector	tertiary sector	With interaction
policy∗tertiary			−0.287^**^
			(0.138)
policy	0.423^***^	0.151	0.448^***^
	(0.124)	(0.136)	(0.123)
covariates	Yes	Yes	Yes
Year	Yes	Yes	Yes
Region	Yes	Yes	Yes
N	3,809	3,175	6,985
$R^{2}$	0.283	0.255	0.214

**, ***Indicates statistical significance at the 5% and 1% levels, respectively.

This pattern reflects differences in employment structures. In traditional sectors such as manufacturing and construction, higher occupational risks and more rigid labor relations make medical insurance more effective in improving job quality by reducing out-of-pocket medical costs. In contrast, the policy effect in the tertiary sector is weaker, possibly due to more flexible employment arrangements and looser institutional constraints. These findings highlight the importance of adapting policy implementation to industry-specific conditions to better support employment quality improvement.

### 4.6 Mechanism analysis

Baseline regression results confirm that the URRMI reform significantly improves employment quality among migrant workers, with evidence of systematic heterogeneity in policy effects. To uncover the underlying transmission pathways, this study draws on the theory of precautionary labor supply and the concept of health-related human capital to construct mediation models testing two primary mechanisms: reduced medical burden and improved health status. Specifically, the medical burden is measured by the logarithm of individual out-of-pocket medical expenses in the past year, excluding any reimbursed or expected-to-be-reimbursed costs. Health status is captured through self-rated health reported by respondents in the survey year.

#### 4.6.1 Medical cost channel

As shown in Table 14, the URRMI reform significantly reduces out-of-pocket medical expenses, which in turn contribute to improved employment quality. The Sobel test confirms the statistical significance of this indirect effect (*Z* = 2.02, *p* < 0.05). These results suggest that lower financial pressure from healthcare reduces the need for precautionary labor behaviors. As medical cost uncertainty declines, migrant workers are better able to avoid excessive overtime or high-risk jobs and instead pursue more stable and quality-oriented employment arrangements.

**Table 14:** Mechanism test.

var	(1) Medical cost			(2) Health status		
	quality	cost	quality	quality	health	quality
quality	0.310^***^	−0.182^***^	0.296^***^	0.310^***^	0.137^**^	0.301^***^
	(0.092)	(0.067)	(0.091)	(0.092)	(0.045)	(0.091)
cost			−0.061^***^			
			(0.009)			
health						0.044^**^
						(0.014)
covariates	Yes	Yes	Yes	Yes	Yes	Yes
Year	Yes	Yes	Yes	Yes	Yes	Yes
Region	Yes	Yes	Yes	Yes	Yes	Yes
N	6,985	6,985	6,985	6,985	6,985	6,985
$R^{2}$	0.260	0.048	0.261	0.260	0.117	0.260

**, ***Indicates statistical significance at the 5% and 1% levels, respectively.

#### 4.6.2 Health status channel

Table 14 also provides evidence of a significant indirect effect via improved health status. The reform enhances self-reported health among migrant workers, which positively affects employment quality. This channel is statistically supported by the Sobel test (*Z* = 2.17, *p* < 0.05). The results highlight the role of public health insurance in enhancing labor capacity by strengthening the physical well-being of the workforce, thereby supporting long-term occupational participation and development.

Although both mediation effects are statistically significant, the direct effect of the URRMI reform remains dominant. The medical cost channel explains approximately 3.55% of the total effect, and the health status channel accounts for 1.94%. This indicates that the majority of the observed impact stems from the direct policy effect, with the two channels playing a supplementary role.

From a policy perspective, these findings underscore the importance of further improving the URRMI system. The medical cost channel highlights the need to optimize reimbursement mechanisms and reduce individual financial burdens, while the health status channel suggests that greater coordination between health services and employment support could enhance overall policy effectiveness. Together, these insights provide a clearer understanding of how integrated health insurance can contribute to improving employment outcomes among migrant workers.

## 5 Conclusion

This study employs a staggered difference-in-differences strategy, combined with propensity score matching to systematically examine the impact of the Urban–Rural Resident Medical Insurance integration on migrant workers’ employment quality. Using four waves of the China Family Panel Studies (CFPS) from 2014 to 2020, along with provincial-level policy implementation records, the analysis incorporates a series of robustness checks to ensure the reliability of the empirical results. Furthermore, heterogeneity analysis and mediation models are employed to uncover subgroup differences and identify underlying transmission mechanisms.

The findings reveal three main conclusions. First, URRMI reform significantly improves the employment quality of migrant workers, with particularly notable effects on wage income, social protection, and job stability. These results suggest that the policy effectively reduces economic uncertainty and enhances access to welfare benefits, thereby improving institutional conditions in the labor market. Second, the policy exhibits considerable heterogeneity across demographic and structural dimensions. In terms of age, workers over 40 benefit more substantially from the reform, likely due to their higher medical needs and greater sensitivity to cost reductions. Regionally, the strongest effects are observed in eastern provinces, highlighting the importance of administrative capacity and healthcare resource availability in shaping policy outcomes. By industry, the policy impact is more pronounced among workers in primary and secondary sectors, where occupational risks are higher and labor relationships more rigid, suggesting stronger incentive effects in these settings. Third, the mechanism analysis identifies two critical pathways: (1) the reform reduces out-of-pocket medical expenses, alleviating liquidity constraints faced by migrant workers; and (2) it improves individual health conditions, contributing to better employment outcomes through enhanced physical capacity and reduced health-related disruptions.

Despite the study’s systematic efforts in empirical identification and mechanism modeling, several limitations remain and warrant future exploration. First, due to constraints in data structure and variable availability, the current construction of the employment quality index adopts a relatively simplified weighting scheme based on conventional practice. It does not fully capture the subjective preferences and multidimensional needs of the migrant worker population. Future research could incorporate subjective assessments and adopt factor analysis or other methods to develop a more inclusive and differentiated evaluation framework. Second, although the mediation analysis focuses on the theoretically grounded channels of medical cost reduction and health improvement, it does not account for more complex dimensions such as healthcare utilization efficiency or mental health. Potential interactions among these factors deserve further investigation. Third, the identification strategy is based on the timing of provincial-level policy implementation, which captures temporal variation in rollout but overlooks within-province variation in local execution. Future research may utilize finer-grained administrative data or policy texts to better capture sub-provincial heterogeneity. Lastly, while CFPS provides nationally representative longitudinal data, it offers limited coverage of marginal labor segments such as informal and highly mobile workers. As such, caution is warranted when generalizing the findings to these groups. Follow-up studies incorporating targeted surveys or qualitative interviews could help evaluate the effects of social protection reforms on the most vulnerable segments of the labor force.

Although this study has made a systematic effort in empirical identification and mechanism modeling, several limitations remain and deserve further attention. First, the employment quality index is mainly constructed using equal weights. While entropy weighting is introduced in the robustness checks to adjust for variation across dimensions, the current measure still does not fully capture the diverse preferences and institutional constraints faced by migrant workers. Future studies should include more subjective indicators and variables related to institutional access to build a more inclusive and informative evaluation framework.

Second, the mechanism analysis mainly focuses on reduced medical burden and improved health status. These channels are supported by both theory and empirical results. However, the mobility of migrant workers and the coordination of health insurance across regions may also influence employment quality. For instance, whether medical expenses can be settled across regions and whether insurance accounts can be transferred smoothly may affect work location decisions, continuity of employment, and vulnerability to health-related risks. These mechanisms are not covered in the current analysis but hold clear policy relevance and should be explored further with appropriate data in future research.

Finally, the identification strategy relies on province-level policy rollout, which captures variation across regions but does not reflect differences in implementation within provinces. Future research may consider more detailed administrative data and policy records to examine within-region heterogeneity. In addition, CFPS provides limited coverage of informal and highly mobile workers. Complementary survey data or interviews may help evaluate how reforms affect these more vulnerable groups.

### 5.1 Policy implications

The findings of this study suggest several policy directions to improve the effectiveness and inclusiveness of the URRMI system.

First, older migrant workers often face greater health risks and employment instability. Improving the accessibility and clarity of reimbursement procedures, and tailoring benefit structures to their specific needs, may strengthen their engagement with the formal welfare system.

Second, regional disparities in implementation remain a concern. Local governments in less-developed areas would benefit from enhanced administrative support and more investment in primary healthcare services, helping ensure that institutional coverage translates into tangible protection.

Third, many migrant workers remain concentrated in labor-intensive sectors where working conditions are rigid. Strengthening the enforcement of labor rights, linking social insurance more closely with occupational protections, and promoting portability across regions could further extend the reform’ s reach.

Finally, establishing a dynamic monitoring system for employment quality—incorporating both objective and subjective indicators—would provide timely feedback on policy outcomes and support data-informed adjustments in future welfare reforms.

These recommendations reflect not only the empirical insights of this study, but also broader challenges in aligning institutional reform with the lived experiences of mobile workers in China’ s evolving urban labor market.

As Amartya Sen stated, “Development is the process of expanding human freedom.” Addressing the employment challenges of migrant workers is not merely a matter of economic efficiency, but a critical issue of social justice and equitable development. Only through the dual drivers of institutional innovation and capacity building can migrant workers truly transition into “new urban citizens,” facilitating a more inclusive and dignified process of urbanization in China.

## Data Availability

Publicly available datasets were analyzed in this study. This study uses data from the China Family Panel Studies (CFPS), a nationally representative longitudinal survey administered by the Institute of Social Science Survey (ISSS) at Peking University. The CFPS database is publicly available to qualified researchers through the official data repository at: http://www.isss.pku.edu.cn/cfps/en/.
